# LOX-1 boosts immunity

**DOI:** 10.18632/oncotarget.4756

**Published:** 2015-07-03

**Authors:** SangKon Oh, HyeMee Joo

**Affiliations:** Baylor Institute for Immunology Research, Live Oak, Dallas, TX, USA

**Keywords:** Immunology and Microbiology Section, Immune response, Immunity

Lectin-like oxidized low-density lipoprotein (LDL) receptor-1 (LOX-1) is a class E scavenger receptor that is encoded by the OLR1 gene on human chromosome 12. LOX-1 is expressed as a type II transmembrane protein containing four domains: an extracellular C-terminal lectin domain, a connecting neck domain, a single transcellular domain and a short N-terminal cytoplasmic tail. A disulfide bond between monomers at cysteine 140 residues results in the formation of a LOX-1 homodimer.

LOX-1 is expressed on the surface of endothelial cells as well as several other cell types, including smooth muscle cells, platelets, and fibroblasts. LOX-1 is also expressed on the surface of other immune cells, such as dendritic cells (DCs) and macrophages. The expression level of LOX-1 can be modulated by inflammatory stimuli as well as by its ligands. Proteolytic cleavage at the neck domain also forms a soluble LOX-1.

Despite lacking the classical signaling motifs, LOX-1 can induce and modulate cell activation via membrane-bound nicotinamide adenine dinucleotide phosphate oxidase followed by the generation of reactive oxygen species that can further activate nuclear factor-κB [1]. Multiple other signaling components, such as phosphoinositide 3-kinase, p38 mitogen-activated protein kinase and protein kinase C, are also known to be involved in LOX-1-induced signaling pathways [1]. LOX-1-mediated internalization of oxidized LDL occurs through a clathrin-independent mechanism involving a novel cytoplasmic tripeptide receptor motif [2]. LOX-1 can thus display multiple cellular functions, including cytokine production and apoptosis-associated cell death as well as endocytosis and phagocytosis.

Due to its expression on vascular endothelial cells and its unique ability to capture oxidized-LDL, C-reactive protein (CRP), and fibronectin, studies on LOX-1 have been mainly focused on its roles in the pathogenesis of vascular diseases (e.g., atherosclerosis). Nonetheless, the roles of LOX-1 in host immune responses remain largely unknown, although one could assume that LOX-1 expressed on antigen presenting cells, particularly DCs, could contribute to host immune responses in either a healthy or diseased condition or in both. This assumption is particularly relevant because DCs are major immune inducers and orchestrators and can thus play key roles in host immunity against cancers and infections as well as in autoimmune diseases.

It was interesting to find that LOX-1 is expressed on CD1c^+^ skin dermal DCs and blood myeloid DCs but not Langerhans cells or plasmacytoid DCs in humans [3, 4]. Not all, but only fractions of peripheral B cells and monocytes also expressed LOX-1 [4]. Furthermore, antigen targeting to DCs via LOX-1 could also efficiently elicit antigen-specific IFNγ-producing CD4^+^ T cell responses both in human *in vitro* and in nonhuman primate *in vivo* [3]. This applied to both naïve and memory CD4^+^ T cell responses that were specific for not only foreign antigen but also self antigen. Our recent data suggest that LOX-1 could have unique functions to promote Th1 responses (unpublished), since Dectin-1 and DC-ASGPR promote Th17 [5] and regulatory T cell responses [3], respectively. The ability to enhance Th1-type T cell responses could be beneficial for host immunity against cancers and viral infections; while such LOX-1-DC-mediated Th1 responses can also be associated with Th1-mediated inflammatory responses.

Histological analysis of human spleens revealed that LOX-1^+^CD11c^+^ DCs interact with IgD^+^ B cells in the marginal zones [4]. This raised a question of whether LOX-1 on the DCs could contribute to B cell responses. This fundamental question was addressed using an agonistic anti-LOX-1 antibody that is specific for human LOX-1. Anti-LOX-1-activated human DCs expressed increased levels of HLA-DR and CD86 and secreted MCP-1, MIP-1α, and IL-8. Most importantly, anti-LOX-1-activated DCs produced BAFF (B cell activating factor) and APRIL (a proliferation inducing ligand), which can promote humoral responses by inducing class switch and by promoting the generation of plasmablasts [6, 7]. No other lectins tested (Dectin-1, DC-ASGPR, DCIR, DC-SIGN, DC-SIGN/L, DEC-205, Langerin, or CLEC6) was able to induce DCs to produce BAFF or APRIL. Therefore, LOX-1 is now considered as an innate receptor that can bridge DCs to B cells to promote humoral immune responses. The ability of anti-LOX-1-activated DCs to promote immunoglobulin class switch, particularly for IgA1 and IgA2, and to imprint CCR10 on plasmablasts [4] provides us with a unique opportunity to design a novel vaccine strategy against mucosal infections. However, it is also noteworthy that LOX-1 expressed on DCs could also play important roles in the pathogenesis of antibody-mediated autoimmune diseases such as lupus. In fact, oxidized-LDL, an endogenous ligand of LOX-1, can also induce DCs to secrete BAFF and APRIL followed by enhanced humoral responses [4].

Taken together, these studies suggested that LOX-1 expressed on human DCs can be a novel target for enhancing cellular and humoral immune responses, while it also serves as a key sensor that can promote inflammatory responses.
